# Brazilin Limits Inflammatory Responses through Induction of Prosurvival Autophagy in Rheumatoid Fibroblast-Like Synoviocytes

**DOI:** 10.1371/journal.pone.0136122

**Published:** 2015-08-21

**Authors:** Hyunji Lee, Seong Wook Kang, Hee Sun Byun, Juhee Jeon, Kyeong Ah Park, Kidong Kang, Wonhyoung Seo, Minho Won, Jeong Ho Seok, Man-Deuk Han, Han-Ming Shen, Gang Min Hur

**Affiliations:** 1 Department of Pharmacology, College of Medicine, Chungnam National University, Daejeon, Republic of Korea; 2 Division of Rheumatology, Department of Internal Medicine, College of Medicine, Chungnam National University, Daejeon, Republic of Korea; 3 Department of Biology, Soonchunhyang University, Asan, Chungnam, Republic of Korea; 4 Department of Physiology, Yong Loo Lin School of Medicine, National University of Singapore, Singapore, Singapore; Toho University School of Medicine, JAPAN

## Abstract

Brazilin is an active compound of *Caesalpinia sappan* L. (Leguminosae), which possesses pro-apoptotic and anti-inflammation potentials depending on the specific cell type. However, it is largely unknown whether autophagy is implicated in the mechanism underlying its chemotherapeutic and anti-inflammatory effects in rheumatoid arthritis (RA). Here, we show that treatment of RA fibroblast-like synoviocytes (FLS) with brazilin results in enhanced level of autophagic flux, evidenced by accumulation of autophagosome and increased level of lipidated LC3 (LC3-II), which is mainly mediated by enhanced production of reactive oxygen species (ROS). Interestingly, long-term exposure of brazilin was able to restore cell survival against the cytotoxity, exclusively in RA FLS, but not in normal fibroblast. Importantly, such a restoration from brazilin-induced cytotoxity in RA FLS was completely abrogated after co-treatment with autophagy inhibitors including NH_4_Cl or chloroquine. Furthermore, we found that the pretreatment of RA FLS with brazilin reduced LPS- or TNF-induced NF-κB activation and the secretion of inflammatory cytokines in parallel with the enhanced autophagic flux. Such anti-NF-κB potentials of brazilin were drastically masked in RA FLS when autophagy was suppressed. These results suggest that brazilin is capable of activating autophagy exclusively in RA FLS, and such inducible autophagy promotes cell survival and limits inflammatory response.

## Introduction

Rheumatoid arthritis (RA) is a common chronic inflammatory disease characterized by inflammation of the synovial lining and progressive destruction of adjacent cartilage and bone [1]. RA synovium displays an unique pathologic feature with diverse cellular populations of monocyte/macrophage (type A) synoviocytes, fibroblast-like (type B) synoviocytes (FLS) and T lymphocytes [2, 3]. Although all populations of cells may contribute to the pathogenesis of disease progression, hyperplastic growth of FLS due to insufficient apoptosis is considered as a pathologic hallmark of RA [4, 5].

Autophagy functions as a highly conserved cellular mechanism to degrade the unnecessary or dysfunctional cellular components through the lysosomal machinery [6]. Considering its important role in cellular homeostasis, dysregulated autophagy has been implicated in the pathogenesis of several diseases including cancer, infection and metabolic disorders as well as autoimmune and/or inflammatory diseases such as systemic lupus erythromatosus (SLE), Crohn disease and RA [7–11]. Although it is still controversial whether autophagy serves as a cell survival or cell death mechanism, the emerging understanding is that autophagy functions as an important survival strategy for cell undergoing stress [12–15]. Of note, autophagy has been shown to be up-regulate in synovial fibroblasts from patients with RA after TNF treatment [16, 17]. RA FLS are capable of producing a large set of inflammatory cytokines and enzymes, and such newly synthesized proteins might be an important mechanism to activate autophagy pathway. Thus it is assumed that, acquisition of resistance to apoptosis during the inflammatory status in RA FLS could be unique cellular phenotype induced by enhanced autophagy to promote survival. Apart from the suspected contribution of deregulated autophagy in anti-apoptotic property in this particular context, autophagy machinery is also known to play an important role in the control of inflammatory and/or immune response [18–20]. Accordingly, genetic or pharmacological inhibition of autophagy has been shown to enhance inflammatory immune response. For instance, mice deficient in autophagy-related molecules such as Atg7 or Beclin-1 showed an excessive inflammation and tissue damage in an experimental arthritis model [21]. Consistently, it has been reported that *in vivo* administration of autophagy activator significantly reduced the level of proinflammatory cytokine in the synovium of mice with arthritis [22]. Thus, promotion of autophagy has therapeutic potential in certain inflammation settings via limiting inflammatory response.

Brazilin [7,11b-dihydrobenz(*b*)indeno(1,2-*d*)pyran-3,6a,9,10(*6H*)-tetrol], the major compound of the traditional herbal medicine *Caesalpinia sappan* L., is a natural red pigment used for histological staining [23]. Brazilin has been known to elicit cellular responses including apoptotic and anti-inflammatory activities, depending on the specific cell type and cellular environment [24–26]. Recently, the extract of dried heartwood of *Caesalpinia sappan* L. and its active compound, brazilin, have been reported to suppress inflammatory responses in vivo arthritis mouse model [27, 28]. The ant-inflammatory properties of brazilin are closely associated with its ability to inhibit the activities of NF-κB or activator protein 1 (AP-1) [25, 29]. Based on its anti-NF-κB potential, earlier studies using brazilin were focused on the transcriptional regulation of inflammatory cytokines mediated by NF-κB following its short-term exposure. However, little is known whether autophagy is implicated in the mechanism underlying its anti-inflammatory effects and its therapeutic efficacy in patients with RA which is characterized by the acquisition of resistance to apoptosis. In this study, upon brazilin treatment, FLS obtained from RA patients exclusively underwent the activation pathway of autophagic flux rather than apoptosis, which functions to resistant to the cytotoxic action of brazilin. Moreover our results provide evidence that the pro-survival autophagy could contribute to anti-inflammatory function of brazilin in RA FLS. Thus our findings provide a novel idea of using brazilin for long-lasting therapy in patients with RA via regulating autophagic process.

## Materials and Methods

### Reagent

Brazilin used in this study was isolated from the dried heartwood of *Caesalpinia sappan L* as described previously [30]. All commercial antibodies and chemicals were purchased from the following resources: Anti-IκB-α, anti-phospho- IκB-α and anti-phospho-p65 antibodies were from Cell Signaling Technology(Beverly, MA, USA); anti-LC3 (L7543), anti-actin(A2066) antibodies, bafilomycin A1 (Baf-A1; B1793), 3-methyladenine (3-MA; M9281), wortmannin (WM; W1628), tert-butyl hydroperoxide (*t*-BHP; 458139), chloroquine (CQ; C6628), N-methyl-N'-nitro-N-nitrosoguanidine (MNNG; 70-25-7), bacterial LPS (from *Escherichia coli*, serotype 0127:B8) and Human pLKO.1 lentiviral contructs from Open Biosystem, targeted Atg5 specific shRNA (NM_004849) were from Sigma-Aldrich (St. Louis, MO, USA). A NF-κB-luc adenoviral vector (Ad5HSV- NF-κB-luc) was a gift from K.C. Sohn (Chungnam National University, Daejeon, Korea). Recombinant mouse TNFα was from R&D Systems (Minneapolis, MN, USA). The caspase inhibitor (z-VAD-FMK; 627610), N-acetyl-L-cysteine (NAC; 106425) and diphenyene iodonium (DPI) were from Calbiochem (San Diego, CA, USA). Mito-TEMPO [(2-(2,2,6,6 tetramethylpiperidin-1-oxyl-4-ylamino)-2-oxoethyl) triphenylphosphonium chloride, monohydrate] (ALX-430–150-M005) and recombinant Fas Ligand (ALX-522-020-C005) were from Enzo Life Sciences (East Farmingdale, NY, USA);

### Ethics Statement

The use of human tissue from knee joint cavity for the generation and culturing of human fibroblast-like synoviocytes (FLS) of RA patients was reviewed and approved by the Ethics Committee of Institutional Review Board (approval number 1012–164) at the Chungnam National University Hospital, Daejeon, Korea. The written informed consent was obtained from each patient by research team prior to surgery.

### Isolation of primary human rheumatoid fibroblast-like synoviocytes (RA FLS) and cell culture

RA FLS were isolated by the enzymatic digestion of synovial tissues obtained from RA patients undergoing total joint replacement surgery or knee synovectomy at Chungnam National University Hospital, Daejeon, Korea. After discarding fat and fibrous tissue, the synovium was minced into small pieces and treated for 2 hours with 2 mg/ml of type II collagenase in HBSS at 37°C in 5% CO2. The tissue was then filtered using fine sterile gauze, washed, and resuspended in DMEM supplemented with 10% FBS, 100U/ml penicillin and 100 μg/ml streptomycin. Dissociated cells were then centrifuged at 800 rpm, following 1,000 rpm and then plated in 10-cm dishes. After overnight culture, the nonadherent cells were removed and the adherent cells were cultivated in DMEM supplemented with 10% FBS, 100U/ml penicillin and 100 μg/ml streptomycin at 37°C in a humidified atmosphere of 95% air and 5% CO2. Primary cultured human dermal fibroblasts (HDF, kindly donated by Dr. J.H. Lee; Chungnam National University, Daejeon Korea), NIH3T3 cells, COS-7 cells and mouse embryonic fibroblasts (MEF) were cultured in in DMEM supplemented with 10% FBS, 100U/ml penicillin and 100 μg/ml streptomycin at 37°C in a humidified atmosphere of 95% air and 5% CO2.

### Lentiviral packaging and knockdown of Atg5 in RA FLS

Short hairpin RNA transduction and lentiviral purification were performed, as described previously [31]. Briefly, recombinant lentiviral particles were produced in HEK293-T cells by co-transfecting lentiviral plasmid containing Atg5 or control shRNA, along with the packaging vectors including pMDL-RRE, pRSV-REV and pVSV-g. After 48h, the supernatants of HEK293-T cells were collected, filtered and concentrated by ultracentrifugation. RAFLS were infected with recombinant virus with the titers of 200 MOI per cells along with 8 μg/ml of polybrene. The cells that were successfully infected by lentiviral particles were selected using 2 μg/ml of puromycin.

### Luciferase reporter assay

NF-κB luciferase assays were performed, as described previously [32]. RA FLS were infected with recombinant adenovirus with the titers of 200 PFU per cells obtained Ad5HSV- NF-κB-luc/PL-DEST-transfected 293A Phoenix packaging cells. Twenty-four hours after infection, cells were treated with LPS (1 μg/ml) for additional 6 h, and luciferase activities in these cells were measured using a luciferase assay kit (Promega, Madison, WI, USA), according to the manufacturer's instructions.

### Determination of cell death

After treatment, as described in the figure legends, cells were trypsinized and collected. Each sample was stained with trypan blue (Bio-Whittaker, 12002–038) and counted with a hemacytometer. The stained cells (blue) were counted as dead cells and were expressed as a percentage of total cells. Data are expressed as the mean ± SE from at least three independent experiments.

### Immunoblot analysis and ELISA analysis

For immunoblot analysis, FLSs were collected and lysed in M2 buffer (20 mM Tris at pH 7.6, 0.5% NP-40, 250 mM NaCl, 3 mM EDTA, 3 mM EGTA, 2 mM DTT, 0.5 mM PMSF, 20 mM ß-glycerol phosphate, 1 mM sodium vanadate, 1 μg/ml leupeptin). Fifty micrograms of the cell lysates were subjected to sodium dodecyl sulfate (SDS) polyacrylamide gel and blotted onto PVDF membrane. After blocking with 5% skim milk in PBST, the membrane was probed with the relevant antibody and visualized by enhanced chemiluminescence (ECL), according to the manufacturer's instruction (Amersham; Piscataway, NJ, USA). ELISAs were used to detect human IL-6 and IL-8 (R&D Systems) in culture supernatant fractions of RA FLS as described previously [30].

### Transmission electron microscopy

After RAS FLS were washed five times with 0.1 M cacodylate buffer containing 0.1% CaCl_2_ at 4°C, cells were fixed with 1% OsO_4_ in 0.1M cacodylate buffer (pH 7.2) containing 0.1% CaCl_2_ for 1 h at 4°C. After rinsing with cold distilled water, cells were dehydrated slowly with an ethanol series and propylene oxide at 4°C. The samples were embedded in EMbed-812 (EMS, 14120). After polymerization of the resin at 60°C for 36 h, serial sections were cut with a diamond knife on an LEICA EM UC6 ultramicrotome (Leica) and mounted on formvar-coated slot grids. Sections were stained with 4% uranyl acetate and lead citrate, and examined under a Tecnai G2 Spirit Twin transmission electron microscope (FEI Company) and a JEM ARM 1300S high voltage electron microscope (JEOL).

### Immunofluorescence analysis

To detect GFP-LC3 translocation, RA FLS cells were grown on glass coverslips and then infected with Ad-GFP-LC3. After 24 h, cells were treated as indicated in the figure legend and fixed with 4% paraformaldehyde for 10 min. Cells were permeabilized with PBS containing 0.2% Triton X-100 and 0.1M glycine and then incubated with DAPI (1 μg/ml) for 15 min. The cells were then washed twice with PBS. Translocation of GFP-LC3 from cytosol to autophagic were imaged under a confocal laser-scanning microscope (LSM5 Live, ZEISS, Thornwood, NY, USA). Quantitative analysis of co-localized fluorescence intensity was measured with the software LSM Image Browser (version 3.5, ZEISS, Thornwood, NY, USA).

### Determination of intracellular ROS production

Production of intracellular ROS was measured using the fluorescent dye 2,7-dichlorofluorescein diacetate (DCF-DA). After treatment, as described in the figure legends, cells were then washed and stained with 1 μM CM-H2DCFDA (Invitrogen Life Technologies, C6827) in Hank’s balanced salt solution (HBSS) for 30 min. Then cells were fixed with 4% paraformaldehyde before collecting cells. The stained cells were analyzed with a FACSCanto II flow cytometer, and data were processed with the FlowJo software (FLOWJO).

### Statistical analysis

Data are expressed as the mean ± SD from at least three separate experiments performed triplicate. The differences between groups were analyzed using a Student’s t-test. *P* < 0.05 were considered statistically significant. Statistical analyses were performed using SPSS software ver. 13.0 (SPSS Inc., Chicago, IL, USA).

## Results

### Restoration from caspase-independent non-apoptotic cell death in RA FLS upon brazilin treatment

**Fig 1:**
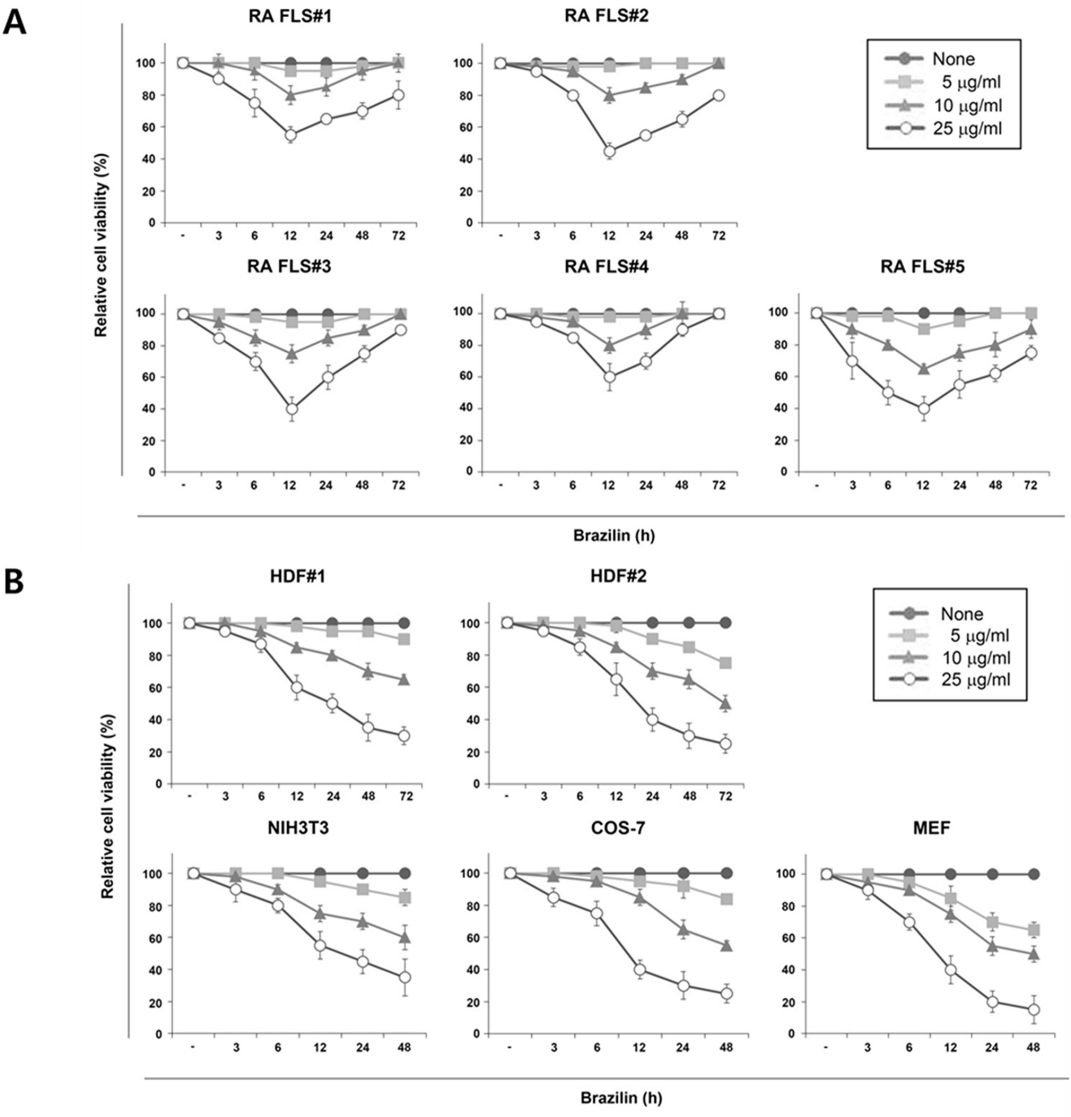
Restoration of cellular survival against from brazilin-induced cytotoxicity in RA FLS, but not in normal fibroblasts. FLS from RA patients (A) and several normal fibroblasts (dermal fibroblast from human foreskin tissues, NIH3T3, COS-7 and MEF) (B) were treated with indicated concentrations of brazilin. The cells were collected and cell viability was then determined by trypan blue exclusion assay. The results are presented as the mean ± S.E. from the three independent experiments.

To investigate the cytotoxicity of RA FLS following treatment with brazilin, time- and dose-dependent cell death was analyzed over a period of 3 days in FLS from 5 RA patients. At the concentrations of 10 and 25 μg/ml brazilin, significant reduction in cell viability were observed by 12 h of treatment in RA FLS (Fig 1A). However, the cytotoxic potentials of brazilin were transient as observed that all RA FLS exclusively showed a restoration of cellular survival after the extended 3-day treatment period. In contrast, under identical conditions, brazilin treatment induced time- and dose-dependent cell death in normal fibroblasts, including primary human dermal fibroblasts (HDF), NIH3T3, COS-7 and mouse embryonic fibroblasts (MEF) (Fig 1B). These results suggest that such an exceptional recovery phenomenon from brazilin-induced cell death in RA FLS is cell type-specific.

**Fig 2:**
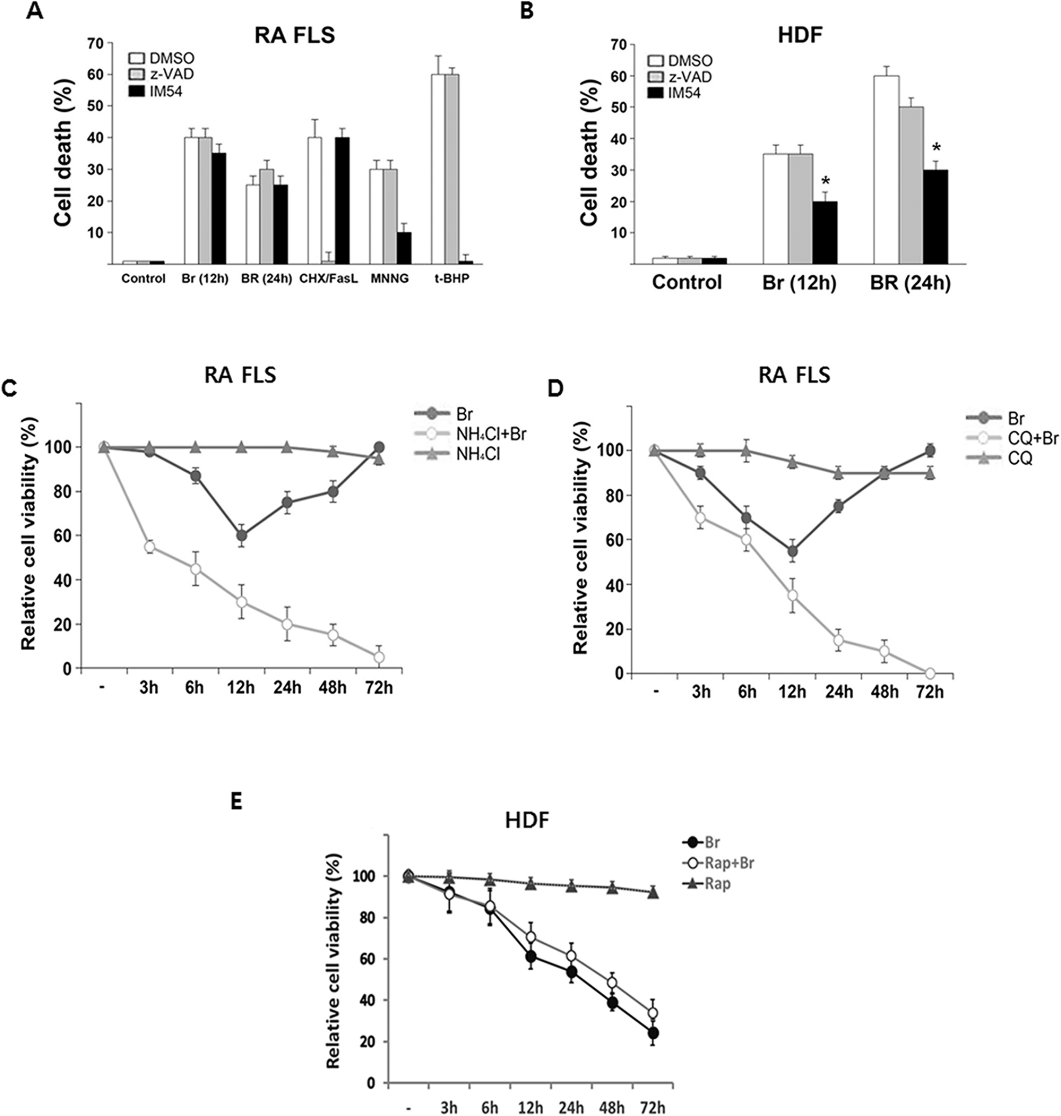
Failure to restoration from brazilin-induced cytotoxicity by blockade of autophagy in RA FLS. (A) RA FLS were treated with brazilin (25 μg/ml), Fas ligand (FasL, 1 μg/ml) plus cycloheximide (CHX, 10 μg/ml), MNNG (0.5 mM) and *t*-BHP (100 μM) in the absence or presence of pancaspase inhibitor (z-VAD-FMK, 20 μM) or necrosis inhibitor (IM54, 10 μM). The cells were collected and cell viability was then determined by trypan blue exclusion assay. The results are presented as the mean ± S.E. from the three independent experiments. (B) HDF were treated with brazilin (25 μg/ml) in the absence or presence of z-VAD-FMK (20 μM) or IM54 (10 μM) as indicated times, and cell viability was determined, as in (A). *P<0.05, compared with brazilin only treated group. (C, D) RA FLS were pretreated with autophagy inhibitor, NH_4_Cl (5 mM) and chloroquine (CQ, 5 mM) for 30 min, and then followed by 25 μg/ml of brazilin as indicated times. (E) HDF were pretreated with rapamycin (100 nM) for 30 min, and then followed by 25 μg/ml of brazilin as indicated times. The cell viability was determined, as describe in Fig 1A.

To characterize the modes of cell death induced by brazilin in RA FLS, we first tested the effect of a pancaspase inhibitor, z-VAD-FMK on the brazilin-induced cytotoxicity. As expected, pretreatment of z-VAD-FMK significantly abrogated apoptotic cell death in response to recombinant FasL/CHX (Fig 2A). However z-VAD-FMK failed to protect the cell from cell death caused by brazilin, indicating that brazilin induces caspase-independent non-apoptotic cell death in RA FLS. Furthermore, pretreatment of a selective inhibitor of necrosis, IM-54 also did not prevent brazilin-induced cell death of RA FLS, whereas the cell death elicited by known necrosis inducer such as MNNG and t-BHP showed a considerable blocking effect by IM-54 (Fig 2A). These results not only indicate that cell death induced by brazilin does not involve necrotic modes in RA FLS but also confirm the effectiveness of IM-54 used in our study. In contrast, IM-54 pretreatment efficiently blocked brazilin-induced cell death in HDF cells (Fig 2B), which suggests that brazilin predominantly triggers necrotic cell death in HDF cells, but not in RA FLS. It also appears that brazilin is able to induce a special form of cell death insensitive to either apoptosis or necrosis inhibitors in RA FLS.

### Enhanced autophagic flux is responsible for RA FLS specific restoration against brazilin-induced cell death

Based on the observations that the brazilin-induced cell death is neither associated with apoptosis nor necrosis, it is assumed that cell death phenomenon as observed above might be autophagy-related. To address this issue, we first explored the impact of pharmacological autophagy blockade on brazilin-induced cell death. Notably, pretreatment of RA FLS with NH_4_Cl, which inhibits the late stages of autophagic process, failed to recover the cell survival from brazilin-induced cell death (Fig 2C). Consistently, such an exceptional restoration phenomena of RA FLS against brazilin-induced cell death was completely disappeared after pretreatment with chloroquin (CQ), another autophagy inhibitor (Fig 2D). No evident cell death was observed when RA FLS were treated with either NH_4_Cl or CQ alone. These results indicate that autophagy is likely to be involved in restoration of cell viability in RA FLS treated with brazilin. However, there was relatively little protection from brazilin-induced cell death in HDF (Fig 2E), HIH3T3, COS-7 and MEF (data not shown) by the pretreatment with autophagy activator rapamycin, suggesting that autophagic process is not involved in the cytotoxic effect of brazilin in normal fibroblasst.

**Fig 3:**
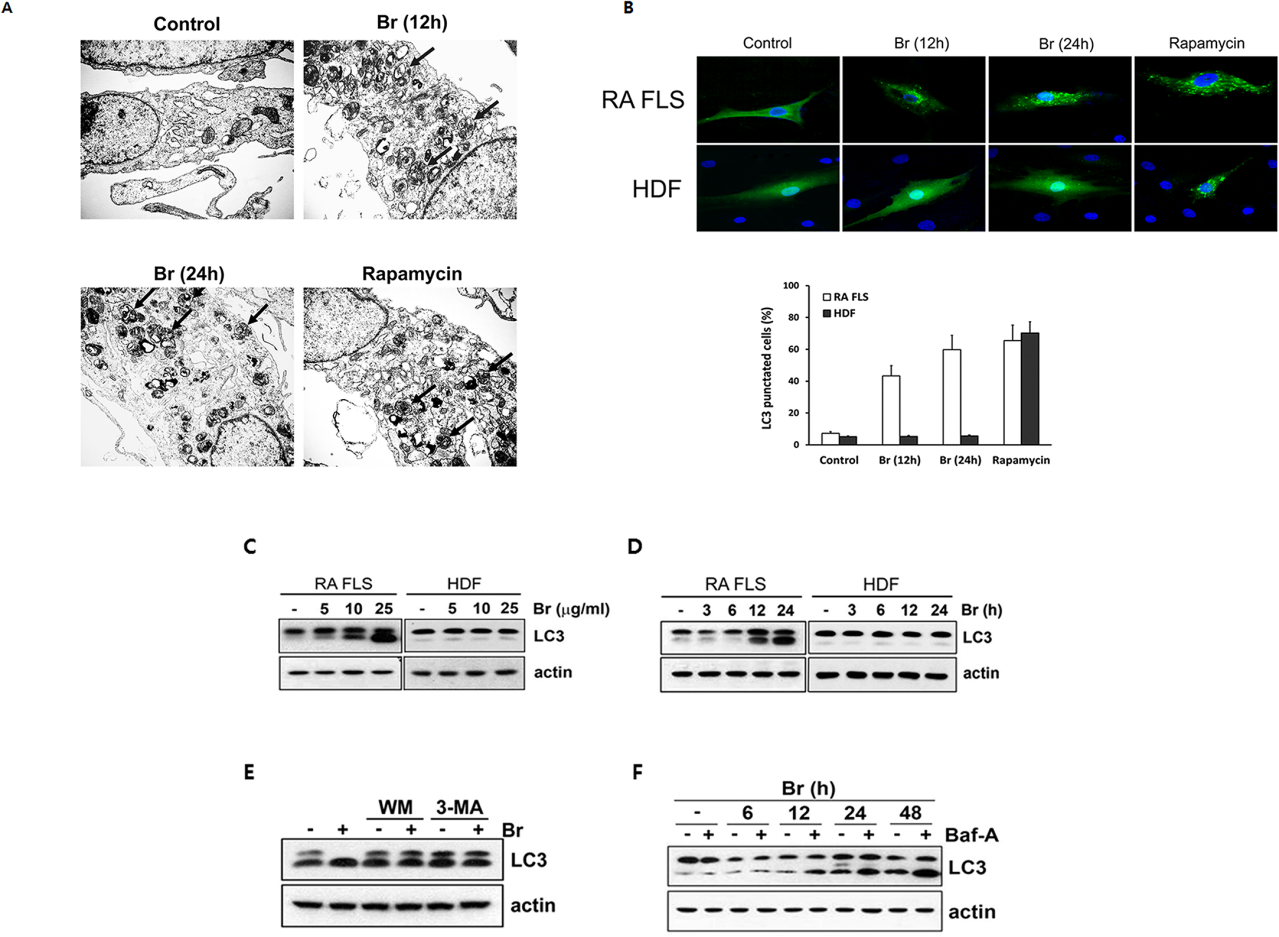
Induction of autophagy in RA FLS in response to brazilin. (A) RA FLS were treated with 25 μg/ml of brazilin or rapamycin (100 nM) for indicated times and imaged by transmission electron microscope (TEM). Representative images of cells are shown. Black arrows indicate accumulation of autophagic vacuoles (autophagosome). (B) RA FLS were infected with recombinant adeno-viral expressing GFP-LC3 at 100 PFU/cells for 2 h, and then replaced with DMEM medium. After 24 h, cells were treated with brazilin (25 μg/ml) and rapamycin (100 nM) as indicated times. Representative fluorescent images of the cells (top). The percentage of cells with GFP-LC3 localized to puntated structures was estimated by counting a minum of 100 cells per sample with values representing the means of triplicate experiments (bottom). (C, D) RA FLS and HDF were treated with brazilin for 24 h as indicated concentrations, and treated with 25 μg/ml of brazilin as indicated times. (E) RA FLS were pretreated with 3-methyladenine (3-MA, 5 mM) or wortmainin (WM, 100 nM) for 30 min, and then followed by brazilin (25 μg/ml) for 24 h. (F) RA FLS were treated with 25 μg/ml of brazilin in the absence or presence of bafilomycin A (BafA, 100 nM). Whole cell lysates were separated by SDS-PAGE and the immunoblotting was performed with anti-LC3 and actin antibodies.

We next explored whether brazilin is capable of inducing autophgy in RA FLS and normal fibroblasts. As shown in Fig 3A, brazilin induced a time-dependent accumulation of numerous lamellar structures with cytosolic autophagic vacuoles, starting at 12 h of treatment, which indicates that brazilin indeed triggers the activation of autophagic process in RA FLS. Rapamycin was used as a positive control (Fig 3A). To compare the effect of brazilin on the autophagic response, RA FLS and HDF were infected with recombinant adenoviral vector carrying GFP-tagged LC3 and examined the distribution pattern of GFP-LC3. Consistently, in RA FLS, brazilin treatment significantly increased the number of cells with high intensity of GFP-LC3 puncta compared to that of untreated control cells. In contrast, brazilin failed to enhance the expression of GFP-LC3 puncta in HDF, whereas rapamycin had the same activating function in both RAFLS and HDF (Fig 3B, top panel). The extent of GFP-LC3 compartments were quantified by counting GFP-LC3 puncta in brazilin- or rapamycin-treated cells (Fig 3B, bottom panel). Furthermore, immunoblotting analysis showed a dose-dependent conversion of the LC3-I to LC3-II starting at 12 h after brazilin treatment in RA FLS but not in HDF (Fig 3C and 3D), confirming that brazilin induces autophagy exclusively in RA FLS. Importantly, the time course of LC3 conversion correlated well with the onset of the recovery of cell survival from the brazilin-induced cell death in RA FLS, as shown in Fig 1A. The enhanced conversion of LC3 was largely abrogated by pretreatment with 3-methyladenine (3-MA) or wortmannin (WM) (Fig 3E), two widely used autophagy inhibitors, which clearly indicating that activation of autophagic process upon brazilin treatment in RA FLS. To exclude the possibility that the results above may arise from blockage of autophagosome turnover, we next assessed the autophagy flux using bafilomycin A (Baf-A), which inhibits the lysosomal degradation of LC3-II [33]. Consistently, in brazilin-treated cells, Baf-A further enhanced the LC3-II level evidently and persistently (Fig 3F), suggesting that brazilin is able to trigger functional autophagic flux in RA FLS.

### Brazilin-induced ROS production is essential for autophagy induction and cell death in RA FLS

**Fig 4:**
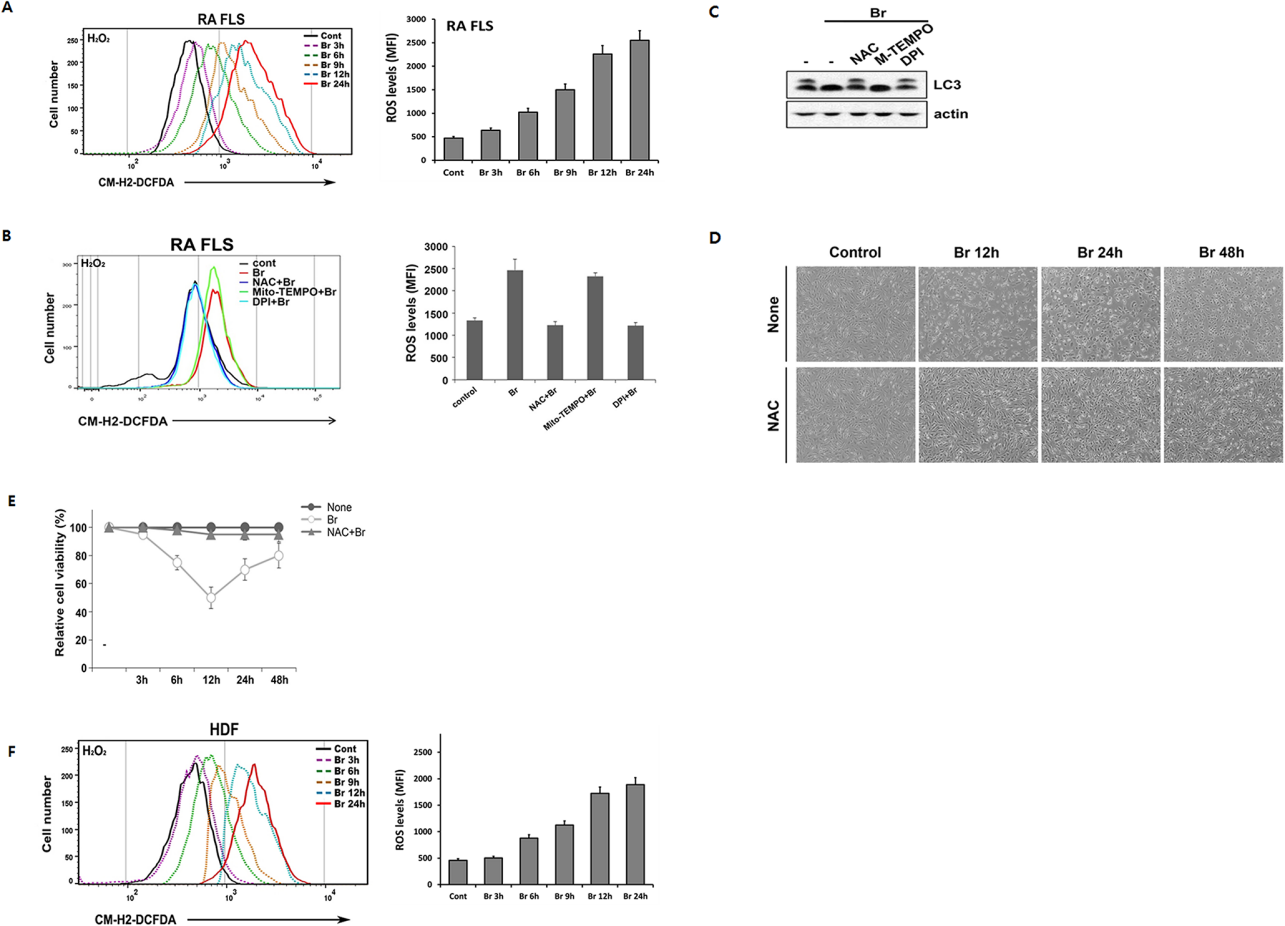
ROS production plays an important role in autophagy activation and early event of cell death in response to brazilin. (A) RA FLS were treated with 25 μg/ml of brazilin for various times, as indicated. CM-H_2_-DCF-DA (1 μM) was added 30 min before end of treatment. ROS were measured with a flow cytometer (left panel) as described in materials and methods. Data were processed and quantified with the FlowJo software (right panel). (B) RA FLS were treated with brazilin (25 μg/ml) in the absence or presence of NAC (10 mM), Mito-TEMPO (100 μM) and DPI (10 μM) for 9 h. Generation of ROS were measured, as in (A) (C) RA FLS were pretreated with NAC (10 mM), Mito-TEMPO (100 μM) and DPI (10 μM), and then followed by brazilin (25 μg/ml) for 24 h. Whole cell lysates were separated by SDS-PAGE and the immunoblotting was performed with anti-LC3 and actin antibodies. (D, E) RA FLS were treated with brazilin (25 μg/ml) in the absence or presence of NAC (10 mM) or Mito-TEMPO (100 μM) for indicated times. Cell were visualized with a normal inverted microscope (D) and the cell viability was determined (E), as describe in Fig 1A. (F) HDF were treated with brazilin (25 μg/ml) for various times, as indicated and the levels of ROS were determined, as in (A).

It has been well established that reactive oxygen species (ROS) promote the induction of autophagy under stress by a number of chemotherapeutics [34, 35]. To see whether intracelluar ROS levels are increased by brazilin, RA FLS were stained with CM-H2 DCFDA, a cell permeable florescent dye that reacts with a broad spectrum of ROS. In different periods of exposure to brazilin, elevated ROS level was detected from 6 h, after which the level gradually continued to increase up to 24 h onwards in RA FLS (Fig 4A). Pretreatment of RA FLS with either general ROS scavenger N-acetyl-L-cysteine (NAC) or the NOX inhibitor diphenyleneiodonium (DPI), but not a specific mitochondria-targeting antioxidant Mito-TEMPO, completely abrogated brazilin-induced ROS production (Fig 4B). We next addressed whether such enhanced ROS production plays a role in autophagy and cell death elicited by brazilin. Blocking ROS production with NAC and DPI, but not Mito-TEMPO, completely inhibited LC3-II conversion (Fig 4C), suggesting that the ROS production, presumably through a NOX family of NADPH oxidase, is required for triggering autophagy in brazilin-treated RA FLS. Furthermore, pretreatment of RA FLS with NAC completely abrogated the early time events of cell death (Fig 4D and 4E). Collectively, data from this part of our study suggest that the activation of autophagy functions as a pro-survival signal against ROS-mediated cell death and responsible for the restoration of cell viability in response to brazilin in RA FLS. As brazilin-induced autophagy activation in RA FLS is cell type-specific, we next examined whether brazilin also affects ROS production in HDF, which failed to induce autophagic responses. Unexpectedly, brazilin treatment of HDF elicited a significant increase in ROS production in a time-dependent manner (Fig 4F), as observed for RA FLS, even though with a lesser extent than in RA FLS. Together with earlier observations that brazilin predominantly triggers necrotic mode of cell death in HDF (Fig 2B), we believe that, in case of HDF, brazilin-induced ROS production contribute to necrotic mode of cell death, rather than triggering the activation of autophagic response.

### Brazilin suppresses NF-κB activation and inflammatory response under the condition of autophagy induction in RA FLS

**Fig 5:**
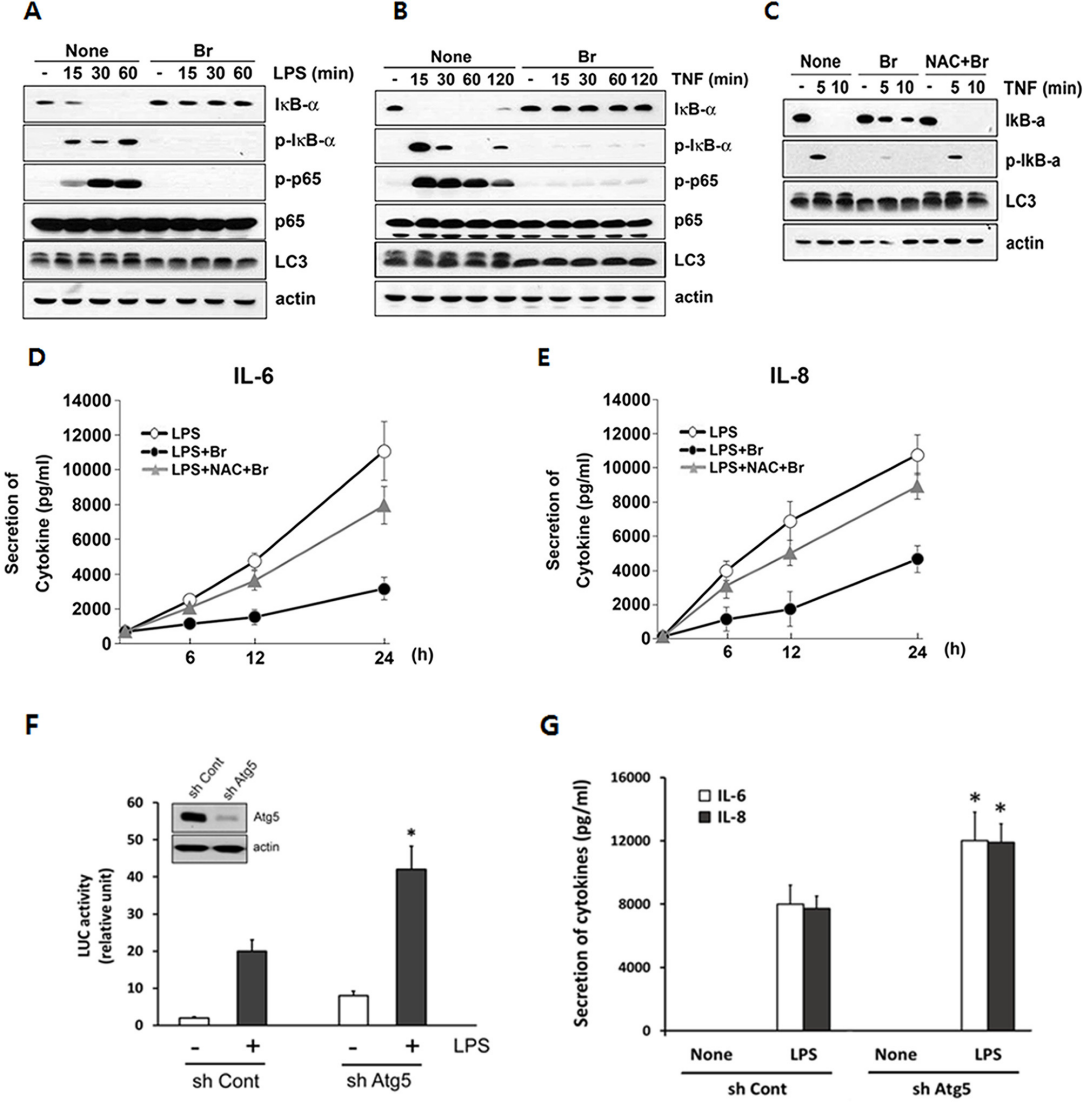
Autophagy induced by brazilin limits NF-κB activation and inflammatory response in RA FLS. (A, B) After pretreatment with brazilin for 48 h, RA FLS were treated with 1 μg/ml of LPS (A) or 15 ng/ml of TNF (B) as for various times as indicated. Whole cell lysates were separated by SDS-PAGE and the immunoblotting was performed using indicated antibodies. (C) RA FLS were pretreated with brazilin for 48 h in the absence or presence of NAC (10 mM), and then treated with TNF as indicated times. Whole cell lysates were separated by SDS-PAGE and the immunoblotting was performed, as in (A). (D, E). RA FLS were pretreated with brazilin for 48 h in the absence or presence of NAC (10 mM). Supernatants were harvested at the indicated times after the commencement of LPS stimulation, and the levels of IL-6 and IL-8 were measured by ELISA. (F) RA FLS transfected with either a lentivirus expressing control shRNA (shCont) or shRNA specific for Atg5 (shAtg5) were cultured for 48 h. Consequently, cells were infected with recombinant adeno-virus expressing NF-κB-Luc at 200 PFU/cells for 2h, and then replaced with DMEM containing 10% FBS. After 24 h of infection. cells were treated for 6 h with LPS (1 μg/ml), and luciferase assay was performed as described in Materials and Methods. Knock down efficiency of Atg5 in RA FLS were assessed by immunoblotting (inset). (G) After RA FLS were transduced with shCont or shAtg5, the cells were further treated with LPS (1 μg/ml) for 24 h. The levels of IL-6 and IL-8 in the supernatant were measured by ELISA. Each columns shows mean ± S.E. from the three independent experiments. *P<0.05, compared with shCont-transfected cells.

Previously, it has been reported that brazilin suppresses inflammatory response through NF-κB signaling pathway in several immune cells [25, 27]. On the other hand, as autophagy is well known to be closely implicated in the regulation of cellular inflammation [18–20, 36, 37], we next assessed the role of autophagy in regulating NF-κB activation and inflammatory response in RA FLS. To address this issue, we pretreated RA FLS with brazilin for 48 h before treatment with either LPS or TNF. LPS or TNF induced rapid phosphorylation and degradation of IκBα as well as p65 phosphorylation (Fig 5A and 5B, left panel). Of note, when the cells were pretreated with brazilin with induction of autophagy evidenced by conversion of LC3-II (Fig 5A and 5B, right panels, fifth rows), LPS and TNF-induced NF-κB activation was drastically inhibited. In contrast, brazilin failed to inhibit TNF-induced phosphorylation and degradation of IκBα (Fig 5C, top and second rows) under the condition of blockade of the ROS-mediated autophagy pathway using NAC (Fig 5C, third row). Consistently, pretreatment of RA FLS with brazilin resulted in a remarkable decrease of the secretion of LPS-induced proinflammatory cytokines IL-6 and IL-8 (Fig 5D and 5E). However, co-treatment with NAC significantly restored LPS-induced IL-6 and IL-8 secretion despite the presence of brazilin. Furthermore, RA FLS transfected with shAtg5 displayed significant increase in NF-κB reporter activity (Fig 5F) and the secretion of IL-6 and IL-8 (Fig 5G) in response to LPS. Such findings support the notion that the activation of autophagy acts as a negative regulator of inflammatory responses through effects on NF-κB, and such an ability of brazilin to enhance autophagy contributes to limit inflammatory responses in RA FLS.

## Discussion

RA is characterized by chronic inflammation of the synovial membrane lining the joint as well as the acquisition of resistance to apoptosis resulting hyperplastic growth of RA FLS, although the underlying mechanism is still not clarified. Having shown a anti-cancer and anti-inflammatory potentials of brazilin in a variety cells including cancer cells and macrophages [24–26, 29], it was assumed that exposure of brazilin in RA FLS may provide an attractive approach for treating RA patients. As expected, brazilin efficiently caused cell death via ROS-dependent necrotic pathway in normal fibroblasts. However, RA FLS exclusively showed a restoration of cellular survival against to the cytotoxity by long-term exposure of brazilin. Important to note, such an extraordinary restoration from brazilin-induced cytotoxity was completely abrogated under the autophagy blockade condition. Moreover, we provide evidence that brazilin enhances autophagic flux in parallel with the suppression of NF-κB activation and inflammatory response. Thus we herein propose that activation of autophagy by brazilin might not only contribute to favour cell survival but also limit inflammatory response in the FLS of patients with RA.

Recently, we have demonstrated that brazilin is a potent NF-κB inhibitor that achieved by targeting IKK or the formation of the upstream IL-1 receptor signaling complex [30]. Given NF-κB plays an essential role in anti-apoptosis, it has been hypothesized that this compound is capable of inducing apoptotic cell death. Indeed, the cancer cells including multiple myeloma and glioblastoma were found a typical apoptotic features with a release of cytochrome c together with activation of caspase-3 and subsequent PARP cleavage [24, 26]. However, we were unable to observe any significant morphological or biochemical characteristics of apoptosis in the tested fibroblasts including RA FLS. Rather, we observed that the transient non-apoptotic or non-necrotic cells death after brazilin treatment until 12 h was unexpectedly vanished by long-term exposure in exclusively RA FLS, but not in normal fibroblast (Fig 1). More importantly, we found that brazilin not only induces drastic autophagic flux but also the onset of autophagy activation correlated well with that the kinetics of the recovery profile from brazilin-induced cell death (Figs 1A and 3). Furthermore, as the unexpected recovery of cell survival was completely abolished under condition of autophagic blockade, it is highly likely that such a cell survival effect by long-term exposure of brazilin is dependent of activation of autophagic process. However, we could not observe any autophagic features, including the punctate LC3 staining and increased levels of lipid-bound LC3-II in normal fibroblast (Fig 3B–3D). Thus we propose that brazilin functions as autophagy inducer which plays a role in favouring survival of RA FLS after its establishment. Nevertheless, how brazilin could induce autophagy exclusively specific to RA FLS is a question that remains yet largely unresolved. As autophagy has been shown to constitutively up-regulated in FLS and osteoclast from RA patients in part mediated by aberrantly un-controlled inflammation-activating signals [16, 17], one possibility is that, in case of RA FLS, the unique cellular context of chronic inflammation in joint from RA patients might be involved in a marked increased sensitivity of autophagic machinery. Therefore, further *in vivo* experiments will be needed to elucidate the tissue specific activation of autophagic process in RA models.

Accumulating evidence has indicated that ROS promote autophagy [34, 35]. In this study, we found that ROS, presumably produced through NADPH oxidase, gradually accumulated after brazilin treatment in RA FLS, and blocking of the ROS production with antioxidant NAC completely blocked the brazilin-induced autophagy (Fig 4), suggest that brazilin-induced ROS production is essential for the induction of autophagy. With regard to kinetics of autophagy induction, we could not observe any biochemical parameter of autophagy activation such as LC-3 conversion within the first 6 h after brazilin treatment (Fig 3D), despite the fact that elevated ROS levels were detectable during this period (Fig 4A). Thus, it is possible that certain levels of ROS accumulation might be required for triggering autophagy in RA FLS. However, it has been reported that, in vascular endothelial cells, brazilin could inhibit high glucose-induced vascular inflammation through inhibiting ROS production [38]. As RA FLS underwent different mode of cell death upon brazilin treatment compared with normal fibroblasts (Fig 2) and cancer cell lines [24–26], these apparent discrepancies with early report [38] might be resulted from tissue-type difference and/or differences in experimental systems (short term exposure *versus* long term exposure of brazilin).

With regards to the functional consequence of autophagy, it has been reported that cells of deficient autophagy machinery has been shown to over-respond to inflammation signals that lead to hyperproduction of proinflammatory cytokines such as TNF and IL-1β [36, 37]. Moreover, some preclinical models suggest that the activation of autophagy could be reduce the severity of experimental arthritis [11]. However, it has not been detailed previously whether autophagy regulates inflammatory responses in primary FLS from RA patients. In this study, we have demonstrated that knockdown of Atg5 enhanced the expression of inflammatory cytokines and NF-κB transcriptional activity in RA FLS (Fig 5F and 5G). Such findings suggest that autophagy induction in primary human RA FLS can negatively regulate inflammatory response. As autophagy is directly involved in the regulation of inflammation, we next examined whether such ROS-mediated autophagy activation might be involved in the anti-inflammatory function of brazilin. In our study, brazilin markedly inhibited the NF-κB activation and the secretion of inflammatory cytokines by LPS or TNF under the condition of long term exposure of brazilin that led to induced sufficient levels of autophagy. Furthermore, such inhibitory roles of brazilin on the NF-κB activation and the secretion of inflammatory cytokines were completely masked by blockade of autophagy activation with antioxidant NAC (Fig 5), suggesting that brazilin-induced autophagy may have a function in limiting NF-κB mediated inflammation process. However, it has been recently proposed that brazilin suppresses the canonical NF-κB activation pathway by either targeting the IL-1 receptor signaling complex formation or the catalytic activity of IKK [30]. One important finding from this study is that brazilin could enhance autophagic flux exclusively in RA FLS, but not in normal fibroblasts. Thus, one possibility of the discrepancy with the recent report is that cell-type specific induction of autophagy may play an important role in dissecting two different anti-NF-κB mechanisms of brazilin: cells harboring abundant autophagy activation such as RA FLS, brazilin may inhibit indirectly NF-κB activation via enhanced autophagy, while in normal or cancer cells which could not induce autophagy, it directly impairs NF-κB signaling pathway. Future studies should elucidate the dynamic correlation between autophagy activation and anti-inflammatory property of brazilin. Also it is important to test the effect of brazilin in actual *in vivo* models to provide more evidence to support the using of brazilin for long-term therapy in patients with RA.
